# Multiple Neuroimaging Measures for Examining Exercise-induced Neuroplasticity in Older Adults: A Quasi-experimental Study

**DOI:** 10.3389/fnagi.2017.00102

**Published:** 2017-04-20

**Authors:** Lanxin Ji, Han Zhang, Guy G. Potter, Yu-Feng Zang, David C. Steffens, Hua Guo, Lihong Wang

**Affiliations:** ^1^Center for Biomedical Imaging Research, Department of Biomedical Engineering, Tsinghua UniversityBeijing, China; ^2^Center for Cognition and Brain Disorders and the Affiliated Hospital, Hangzhou Normal UniversityHangzhou, China; ^3^Zhejiang Key Laboratory for Research in Assessment of Cognitive ImpairmentsHangzhou, China; ^4^Brain Imaging and Analysis Center, Duke University Medical Center, DurhamNC, USA; ^5^Department of Psychiatry, University of Connecticut Health Center, FarmingtonCT, USA

**Keywords:** physical exercise, neuroplasticity, aging, cognitive function, resting state functional connectivity, multiple imaging modality

## Abstract

Physical exercise can improve physical and mental health. A number of imaging studies have examined the role of neuroplasticity in improving cognition with physical exercise; however, such neuroplasticity changes are not consistent across the reports partly due to small sample sizes in some studies. We thought to explore the concept that identifying consistent findings across multi-modality imaging measures would provide relatively reliable results. We designed a 6-week quasi-experiment with Wii-fitness exercise program in 24 healthy adults older than 60, and then examined the changes on neuroimaging measures including brain volume, the amplitude of low-frequency oscillation function (ALFF), regional homogeneity (ReHo), seed-based functional connectivity (FC), and the global efficiency of nodal connectivity during resting state. We focused on whether there were common regions showing changes after exercise across these measures and which measure was closely correlated with cognitive improvement. After the six-week exercise program, participants demonstrated a significant improvement in memory and executive function on neuropsychological tests, and in memory recall on an emotional memory task. The common brain regions that showed significant changes across different measures were the right striatum and the posterior cingulate (PCC). After exercise, the PCC showed decreased ReHo and increased volume, and the striatum did not show volume loss as the control group did and increased its FC with the cingulate, temporal, parietal, and occipital regions. Moreover, the connectivity change between the striatum and the thalamus was correlated with the improvement of executive function. This result implicates the striatum and the PCC associated network in physical exercise. Our work highlights the effectiveness of multi-modality neuroimaging measures in investigating neuroplasticity.

## Introduction

Lack of physical exercise has been found to be one of the major contributors to development of Alzheimer disease (Barnes and Yaffe, 2011). Therefore, using physical exercise to improve cognitive function in older adults has gained increasing attention in the field of neuroscience research in recent years. There is abundant evidence in the literature showing that physical exercise not only can improve cardiovascular function, but also can improve cognitive function including speed, visual spatial, executive and cognitive control processing (Colcombe and Kramer, 2003; Lautenschlager et al., 2009; Barber et al., 2012). Neuroimaging research has been extensively conducted to investigate the training-induced neuroplasticity changes in the brain, however, thus far no consistent findings are found across reports partly due to the discrepancy of neuroimaging measures used among the studies in the literature (Colcombe et al., 2006; Boyke et al., 2008; Lustig et al., 2009). Studies measuring neuroplasticity change varied from brain volume, regional task-related activations, and perfusion change, to brain activity and functional connectivity (FC) during resting state, such as seed-based FC (Biswal et al., 1995), the amplitude of low-frequence fluctuation (ALFF, Zang et al., 2007), regional homogenity (ReHo, Zang et al., 2004), and graph theory analysis (Bullmore and Sporns, 2009). Functional connectivity and graph theory analysis have been widely used to measure correlations between spatially distinct brain areas. The ALFF of the resting-state fMRI signal has been suggested to reflect the intensity of regional spontaneous brain activity, while Reho is proposed to describe regional homogeneity of neural activities. While exercise-induced neuroplasticity changes in each of these neuroimaging measures have been reported previously, there are very few to integrate these methods together and investigate the common findings across the measures and compare which one is more closely correlated with changes in cognition.

Comparing the association between the changes of different neuroimaging measures with cognitive improvement would provide useful guidance for future imaging studies in exercise-induced neuroplasticity. Importantly, converging the findings from multiple neuroimaging measures would provide more reliable results than a single measure. Given the difficulty in executing exercise-intervention trial studies, some of the published exercise-related MRI studies in the literature have enrolled a small number of participants (Colcombe et al., 2006; Burdette et al., 2010; Holzschneider et al., 2012), which challenge the reliability of the reported results. We reason that identifying the common regions that show changes across different imaging modalities could be a reasonable solution for providing reliable results in studies with small sample sizes. Although each of these parameters reflects neuroplasticity with different neural mechanisms, those regions that repeatedly show changes in different types of measurements might have truly been shaped by physical exercises. To our best knowledge, there is only one cross-sectional study (Di et al., 2012) that has combined different imaging modalities which demonstrated that professional badminton training increased gray matter (GM) volume and the strength of neural oscillation (measured by ALFF) in the cerebellar regions. There are no longitudinal studies so far that have compared how consistent different neuroimaging measures are in reflecting neuroplasticity. Therefore, in this study, we aimed to investigate the consistent changes induced by physical exercises across different neuroimaging measures, and examine which measure is more closely correlated with cognitive improvement.

Previous studies have shown that participants who were in combined strength and aerobic training regimens improved cognitive function to a reliably greater degree than those who had aerobic training alone (Colcombe and Kramer, 2003). We posited that exercise programs in multiple domains that require the coordination of multiple neural systems to complete the exercise program might induce stronger neural plastic change than exercise in a single domain. Therefore, we designed a 6-week Wii-fitness exercise training program including aerobic, balance, weight lifting, and yoga. We expected this multi-domain exercise regimen would have a robust effect on cognitive function. The impact of exercise was evaluated on several imaging measurements including the brain GM volume, resting state low-frequency fluctuation activity, homogeneity, FC, as well as global connectivity efficiency of nodal networks in older adults. We hypothesized that motor and motor skill related regions such as striatum, motor cortex, and cerebellum, as well as attention and executive function related regions such as prefrontal cortex would reveal plasticity changes post exercise commonly in different imaging modalities. The plasticity changes in these regions may be associated with cognitive improvement after physical exercises.

## Materials and Methods

### Participants

Twenty-four healthy subjects (70 ± 7.78 years; 12 females) were recruited through advertisements. Two subjects in the control group were left hand dominant, and the rest were right hand dominant. Exclusion criteria for the study were: (1) MRI contraindications and claustrophobia; (2) severe or unstable medical disorders, conditions, or drugs that may cause any condition that in the investigators’ opinion might make the patients unsuitable for participating in the study (e.g., clinically significant cirrhosis, or heart disease); (3) any known current or past diagnosis of psychiatric disorders; (4) active suicidality or current suicidal risk as determined by the investigator; (5) significant handicaps that would interfere with neuropsychological testing or the inability to follow study procedures; (6) any known primary neurological disorders such as tumors, multiple sclerosis, or seizure disorder; (7) Mini-Mental State Examination (MMSE) <24; (8) any repetitive motion injury (e.g., tendinitis, bursitis, etc.); (9) extreme upper extremity arthritis; and (10) any other factor that in the investigators judgment may affect patient’s safety or compliance. In addition, all subjects completed a demographic data form and a detailed questionnaire to screen out individuals who may be at-risk of injury during physical exercise. Subjects were also administered a neuropsychological testing battery (see the Assessments on Cognitive and Psychological Function below). Subjects who had performance below 2 SD in any two tests of each cognitive domain (memory, executive function, or information processing speed) were removed from the study to ensure the cognitive status of all subjects being in normal range. The study received ethics committee approval by the Duke University School of Medicine Institutional Review Board. All subjects gave their written consents after being explained the purpose and procedures prior to the study.

### Exercise Training and Experimental Procedure

The study was a quasi-experiment. The first four subjects were all controls to ensure the protocol work well. The rest of participants were randomly assigned by a research assistant either to participate in physical exercise training (Wii fit, Nintendo) (*n* = 12; five females; 67±6.40 years old) or to be on the no-training waiting list serving as a control group (*n* = 12; seven females; 73 ± 8 years old). Detailed demographic profiles of participants are summarized in **Table 1**. The exercise training program contained exercises in four domains, i.e., aerobic, balance, weight lifting, and yoga. A research assistant helped the participants to set up the Wii device and taught them how to do the Wii exercises. Participants were instructed to practice at home 30 min every day for 6 weeks. The Nintendo Wii system recorded exercise types, time, and duration automatically. A research assistant visited participants weekly to monitor the compliance and changes in mood. The mood state was evaluated by the Positive and Negative Affect Schedule (PANAS). One participant did not complete the PANAS form.

**Table 1:** The demographic profile of participants and cognitive function at baseline.

	Control group (*N* = 12)	Training group (*N* = 12)	*p*-value
Age (years)	73.0 (8.00)	67.0 (6.40)	0.053
Sex (M/F)	5/7	7/5	0.684
Dominant hand (R/L)	10/2	12/0	0.481
Education (years)	16.3 (2.56)	17.2 (2.09)	0.35
Montgomery-Asberg Depression Scale	0.67 (1.30)	0.55 (1.29)	0.820
Memory Function	–0.03 (0.23)	0.17 (0.26)	0.58
*RM_Imm (z)*	0.35 (0.24)	0.22 (0.52)	0.81
*RM_Delay (z)*	0.34 (0.24)	0.22 (0.60)	0.85
*HVLT_Imm (z)*	–0.08 (0.25)	0.32 (0.26)	0.29
*HVLT_Delay (z)*	–0.28 (0.34)	0.20 (0.27)	0.30
*HVLT_Recog (z)*	–0.49 (0.34)	–0.01(0.26)	0.31
Executive Function	0.03 (0.23)	0.04 (0.28)	0.99
*Trails B Making Test (z)*	0.53 (0.32)	0.41 (0.43)	0.82
*Stroop Color and Word Test (z)*	–0.64 (0.21)	–0.34 (0.21)	0.32
Working Memory Function	–0.11 (0.24)	0.37 (0.32)	0.24
*WAIS-III Digit Span (z)*	–0.11 (0.24)	0.37 (0.32)	0.24
Information Processing Speed	0.74 (0.15)	0.78 (0.31)	0.89
*DSST (z)*	1.22 (0.19)	1.30 (0.36)	0.85
*Trails A Making Test (z)*	0.25 (0.23)	0.27 (0.39)	0.96

*RM_Imm, RM_Delay: Immediate and Delayed Story Recall from the Rivermead Behavioral Memory Test. HVLT_Imm, HVLT_Delay and HVLT_Recog: Immediate, Delayed and Recognition Recall from Hopkins Verbal Learning Test-Revised DSST: WAIS-III Digit-Symbol Substitution Modality Test.*

### Assessments on Cognitive and Psychological Function

A neuropsychological testing battery was used to assess cognitive function. The battery included Immediate, Delayed, and Recognition Recall from Hopkins Verbal Learning Test-Revised (HVLT_Imm, HVLT_Delay and HVLT_Recog), Immediate and Delayed Story Recall from the Rivermead Behavioral Memory Test (RM_Imm and RM_Delay), WAIS-III Digit-Symbol Substitution Modality Test (DSST), WAIS-III Digit span, Trail Making Test (Trails A and Trails B), and Stroop Color and Word Test. All testing items were converted to *z* scores based on subjects’ age, gender, race, and education level. We operationalized memory function using HVLT_Imm, HVLT_Delay, HVLT_Recog, RM_Imm and RM_Delay tests; executive function using Trails B and Stroop Color and Word Tests; working memory function using Digit Span; and information processing speed using the DSST and Trails A tests. The physical exercise training group completed the cognitive assessment both before and after the 6-week training. The control group only completed the initial tests on this battery, but not the retest after 6 weeks due to the failure of attempting to identify a second version of comparable neuropsychological testing battery. Since we did not examine the test–retest effect for the neuropsychological testing battery in this control group, we cannot rule out the possibility that significant improvement that we would find in cognitive function after physical exercise are due to a learning effect. Therefore, we further conducted a computerized memory task during the fMRI scan. The details of the task are introduced in the image acquisition section. There was one subject in the control group and two subjects in the physical exercise training group who didn’t complete the memory task. As mentioned earlier, the PNANS was used to assess mood state each week after exercise.

### Imaging Acquisition

MRI scans were conducted at the Brain Imaging and Analysis Center on a 3.0 T GE EXCITE HD scanner (GE Medical Systems, Milwaukee, WI, USA). During each neuroimaging session, the following image acquisition protocols were used. First, sagittal T1-weighted spin-echo images were collected to identify landmarks for reference. Second, a T1-weighted 3D-SPGR sequence provided high-resolution anatomical images with 216 slices, slice thickness = 1 mm, acquisition matrix = 256 × 216 × 256, FOV (field of view) = 256 mm, TR (repetition time) = 7 ms, TE (echo time) = 3 ms, flip angle = 12°. Third, a 5-min resting-state fMRI scan was acquired using the following parameters: TR = 2000 ms, TE = 31 ms, FOV = 240 mm, flip angle = 90°, matrix = 64 × 64 × 34, slice thickness = 3.75 mm. Subjects were instructed to focus on a cross sign which was presented in the center of the screen. Fourth, a five-run emotional memory task-related fMRI scan was acquired. Three hours prior to the fMRI scan, subjects learned a set of happy, sad, and emotionally neutral pictures. They were asked to label the emotional category of the pictures and describe the details of the picture so that to remember these pictures. During the fMRI scan, the old pictures (learnt 3 h prior) as well as new pictures were presented randomly. In order to test immediate memory recall, half of the new pictures were also repeated (0–2 back). Subjects were asked to identify whether the pictures were old, new, or repeated pictures by pressing one of the three different buttons. The pictures used in the emotional memory task before (visit 1) and after (visit 2) 6 weeks were different to avoid learning effect. Given that we were only interested in comparing resting-state fMRI measures, we did not analyze the data during the emotional memory fMRI task in this report. Rather, the task was served as a behavior measure and only the results of memory recall performance are reported in this study.

### MRI Structural Data Preprocessing and Analyses

The T1-weighted structural images were preprocessed using SPM8^1^ running under the Matlab2012b environment. The images were initially preprocessed using VBM8 Toolbox^2^ implemented in SPM8 and this involved segmentation, registration to the standard Montreal Neurological Institute (MNI) space and modulated normalization. A study-specific GM template was created from the preprocessed GM segments of all subjects using Template-O-Matic (TOM8) toolbox^3^. Then the spatially normalized GM segments were warped to the study-specific template and “modulated” by the Jacobian determinants of the deformations to account for local compression and expansion due to linear and non-linear transformation. Finally, the modulated GM volumes were smoothed with a Gaussian kernel of 8 mm full width at half maximum (FWHM) (Draganski et al., 2011). One participant in the control group was excluded from the analysis due to the damage of images.

The students’ two sample *t*-test was applied to examine significant differences between the two groups on the changes of the VBM map (visit 2 – visit 1) with age and sex being controlled. The threshold of significance was set at *p* < 0.05 using alphasim cluster correction [cluster size >389, connection criteria (rmm) = 5]. The alphasim correction was conducted on REST analysis toolkit (Song et al., 2011).

### Resting State fMRI Data Preprocessing and Analyses

The resting state fMRI data was preprocessed using DPARSF^4^ and SPM8 Toolbox^5^. The pre-processing steps included slice timing, realignment, spatial co-registration to each participant’s own T1 image and then warping to the MNI space according to the deformation field information generated in structural T1 image processing, and resampled to 3 mm × 3 mm × 3 mm in voxel size. One participant in the physical exercise training group had head motion of more than 2.0 mm displacement or 2.0 degrees in maximum and was excluded. One subject in the control group was excluded because of damage to the T1 image.

Both the ALFF and ReHo analyses were conducted using DPARSF^2^. For ALFF (Zang et al., 2007), filtered time series (0.01–0.08 Hz) was transformed to a frequency domain with a fast Fourier transform (FFT) and the power spectrum was then obtained. The square root was thus calculated at each frequency of the power spectrum and the averaged square root was obtained across 0.01–0.08 Hz at each voxel. This averaged square root was taken as the ALFF.

The ReHo value was calculated to measure the similarity of the time series of a given voxel to its nearest 26 voxels (Zang et al., 2004). Through calculating the ReHo value of every voxel in the whole brain, individual ReHo maps were generated. Finally, the ALFF and ReHo maps were smoothed with a Gaussian kernel of 8 mm FWHM. To reduce the influence of individual variations, normalization of ReHo maps and ALFF was preformed through converting the maps to *z*-scores by subtracting the whole brain average ReHo/ALFF value and then dividing by the standard deviation of ReHo/ALFF values across all the voxels in the brain.

To examine the differences in the changes of ALFF and ReHo after 6 weeks between the physical exercise training group and control group, we first subtracted the ALFF and ReHo of visit 2 from visit 1 for each subject. Then the student‘s two-sample *t*-test was performed on the voxel-based difference maps of the two groups to calculate group difference in ALFF/ReHo changes. Age and gender effects were regressed out during the two-sample tests. Voxels with a *p*-value < 0.05 (corrected by AlphaSim, as implemented in the REST, with the following parameters: *p*-value at single voxel = 0.05, connection criteria (rmm) = 5, cluster size >389) were considered as having significant difference between the two groups.

To examine an effect of training on FC, pair-wise correlation analyses were conducted. For each participant, the Automated Anatomical Labeling template (AAL, Tzourio-Mazoyer et al., 2002) was used to divide the entire brain into 116 regions (including the cerebellum) as regions of interest. The mean time courses were extracted from each region and used to obtain a 116 × 116 correlation matrix of Pearson’s correlation coefficients. This resulted in a 116×116 correlation matrix with 6670 [(116 × 115)/2] unique inter-regional correlation coefficients (*r*). Then we performed 6670 separate students’ two sample *t*-tests on the difference of two visits (visit 2 – visit 1 within subjects). Multiple comparisons were corrected using a degree-based correction based on non-random data distribution patterns proposed by Chou et al. (2015) with 325069 ([61^∗^73^∗^61]) voxels. The regions which had at least 15 functional links (out of 116 × 116 matrix) connected to other regions with a significance of *p*<0.05 were considered significant.

We also evaluated the whole-brain FC changes at the network level for each node using the graph theory method. Based on the generated 116 × 116 correlation matrix as described above, we evaluated the following network properties (Burdette et al., 2010) of each subject using GRETNA toolbox^6^: (1) Nodal degree: A measure of connectivity to other regions of each node and (2) Global efficiency: A measure of the closeness of an individual node to all other nodes.

To confirm that there was no significant difference at baseline, we conducted two-sample *t*-tests on all neuroimaging measures.

### Correlation between the Neuroimaging Measures and Cognitive Function

We have extracted those regions that have demonstrated a significant between-group difference for each measure as ROIs, and conducted ROI analyses to further examine whether the changes of our tested measures in those regions were correlated with any changes in cognitive function at each domain. Pearson’s correlation analysis between neuroimaging measures with the four domains of cognitive measures was conducted separately. The threshold of significance was set at *p*<0.01 (0.05/4 cognition domains) to correct multiple comparisons.

## Results

### Changes in Cognitive Function after the Exercise Training

**Table 1** provides demographic and cognitive comparisons of the two groups at baseline. No significant differences were found between the two groups in any of the demographic profiles. There was no significant difference between the two groups in cognitive performance at baseline either (**Table 1**).

**FIGURE 1:**
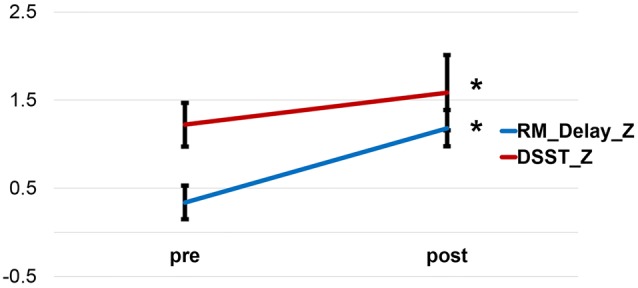
**Improvement of the Delayed Story Recall Test and the Digit-Symbol Substitution Test after a 6-week exercise training.** RM_Delay, Delayed Story Recall Test. DSST, Digit-Symbol Substitution Test.

We examined the changes of neuropsychological tests before and after the physical exercise training in the exercise training group. All neuropsychological tests scores increased after exercise, except for HVLT_Recognition. This group demonstrated a significant improvement in memory during the Delayed Story Recall from the Rivermead Behavior Memory Test (RM_Delay) (*p* = 0.017) and in executive function during the WAIS-III Digit-Symbol Substitution Modality Test (DSST) (*p* < 0.001) after relative to before the 6-week exercise training (paired *t*-test) (**Figure 1**). However, after familywise error (FWE) correction for multiple comparisons, only DSST remained significant. **Table 2** provides the detailed descriptive statistics for the neuropsychological tests and mood measurements. Regarding participants’ performance on the memory task during fMRI scan, the discrimination rate (i.e., Hit-False alarm rate) was used to assess the accuracy of recognition memory. We conducted two sample *t*-tests to compare the exercise training group with the control group in the improvement of the discrimination rate after vs. before 6 weeks (visit 2 - visit 1). The exercise training group showed significantly greater improvement in the discrimination rate than the control group (*p* = 0.0313). However, two subjects in the exercise training group had very low discrimination rate at their visit 1, and we speculated that they might have been somnolent during the scan. Given the low discrimination rate of the two subjects, the distribution was skewed. We further conducted the non-parameter Mann–Whitney test. The Mann–Whitney test confirmed that the discriminate rate of the exercise-training group increased significantly (*p* = 0.035) compared with the control group after versus before 6 weeks.

**Table 2:** Descriptive statistics for the neuropsychological testing data and mood measurements before and after exercise.

	Pre training	Post training	*p*-value
	Mean (*SD*)	Mean (*SD*)	
**Cognitive function**			
Averaged *z*-score of all tests	0.12 (0.17)	0.32 (0.21)	0.147
Memory Function	–0.03 (0.23)	0.20 (0.35)	0.353
*RM_Imm (z)*	0.35 (0.24)	0.88 (0.41)	0.153
*RM_Delay(z)*	0.34 (0.24)	1.18 (0.43)	0.017ˆ*
*HVLT_Imm (z)*	–0.08 (0.25)	0.02 (0.28)	0.648
*HVLT_Delay (z)*	–0.28 (0.34)	–0.06 (0.42)	0.457
*HVLT_Recog (z)*	–0.49 (0.34)	–1.04 (0.38)	0.132
Executive Function	0.03 (0.23)	0.23 (0.21)	0.168
*Trails B Making Test (z)*	0.53 (0.32)	0.75 (0.24)	0.35
*Stroop Color and Word Test (z)*	–0.64 (0.21)	–0.52 (0.25)	0.464
Working Memory Function	–0.11 (0.24)	0.06 (0.20)	0.237
*WAIS-III Digit Span (z)*	–0.11 (0.24)	0.06 (0.21)	0.237
Information Processing Speed	0.74 (0.15)	0.93 (0.19)	0.092
*DSST (z)*	1.22 (0.19)	1.58 (0.21)	<0.001ˆ*
*Trails A Making Test (z)*	0.25 (0.23)	0.27 (0.27)	0.938
Memory task (Hit-False alarm rate)	–0.01 (<0.01)	0.21 (0.03)	0.031ˆ*
**Mood state**			
PANAS_Positive	34.09 (1.85)	33.91 (1.80)	0.824
PANAS_Negative	11.27 (0.36)	11.09 (0.46)	0.774

*^∗^Paired *t-*test, *p* < 0.05. RM_Imm, RM_Delay: Immediate and Delayed Story Recall from the Rivermead Behavioral Memory Test. HVLT_Imm, HVLT_Delay and HVLT_Recog: Immediate, Delayed and Recognition Recall from Hopkins Verbal Learning Test-Revised. DSST, WAIS-III Digit-Symbol Substitution Modality Test. PANAS, Positive and Negative Affect Schedule.*

### Gray Matter Volumetric Changes after the Exercise Training

**FIGURE 2:**
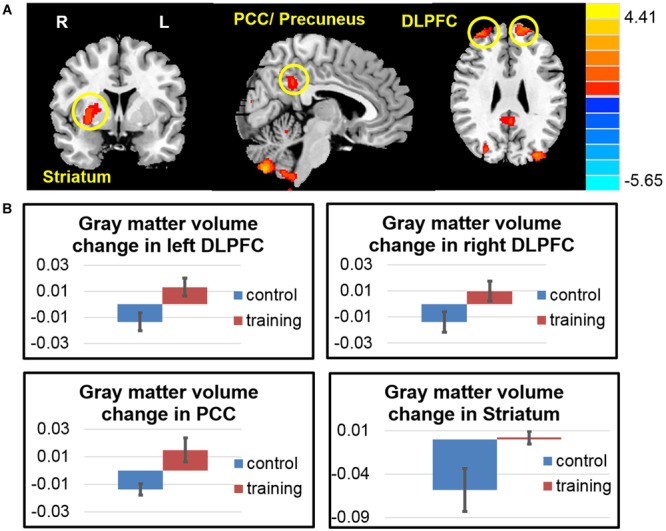
**Gray matter volume changes after exercise training. (A)** The exercise training group had significantly increased gray matter volume in the dorsolateral prefrontal cortex (DLPFC), posterior cingulate cortex (PCC), striatum, hand motor area, occipital lobe and cerebellum. **(B)** Averaged gray matter intensity change in the regions of interest among two groups.

The volumetric changes using VBM revealed that, compared with the control group, the exercise training group had significantly increased GM volume in the dorsolateral prefrontal cortex (DLPFC), posterior cingulate (PCC)/precuneus cortex, hand motor area, occipital lobe, and cerebellum (**Figure 2**). And the striatum did not show volume loss as the control group did. Detailed results are summarized in **Table 3**.

**Table 3:** Regions increased gray matter volume after 6 weeks (visit 2 – visit 1) in the exercise group compared with the control group.

Region	Hemisphere	Cluster size	Peak MNI coordinates			Peak intensity (t)
			*x*	*y*	*z*	
Dorsolateral prefrontal cortex (DLPFC)	Left	904	–13.5	58.5	36	4.4129
Dorsolateral prefrontal cortex (DLFPC)	Right	1077	37.5	54	25.5	3.8444
Striatum (putamen)	Right	590	27	–1.5	–4.5	3.2625
Posterior cingulate / Precuneus cortex (PCC)	Right	904	6	–48	34.5	2.6374

### Functional Changes after the Exercise Training

**FIGURE 3:**
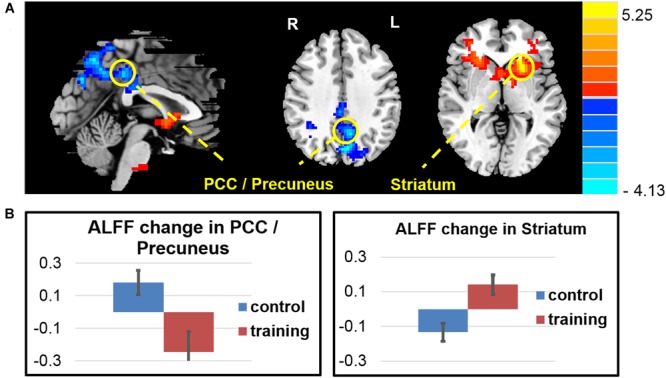
**Amplitude of low-frequency oscillation function (ALFF) changes after exercise training. (A)** The exercise training-induced ALFF reduction in the PCC/precuneus area and ALFF increase in the left striatum extended to left anterior insula, right anterior insula, right caudate, right entorhinal cortex, and left cerebellar cortex. **(B)** Averaged ALFF *z*-score change of two groups in the regions of interest.

The whole brain voxel-wise ALFF analysis revealed that the exercise training decreased ALFF in the PCC/precuneus cortex and increased ALFF in the left striatum extended to the anterior insula, right anterior insula, right caudate, right entorhinal cortex and left cerebellar cortex (**Figure 3** and **Table 4**) compared with the control group (two-sample *t*-test on the difference of two visits, *p* < 0.05, AlphaSim correction).

**Table 4:** Regions changed ALFF after 6 weeks (visit 2 – visit 1) in the exercise group compared with the control group.

Region	Hemisphere	Cluster size	Peak intensity	Peak MNI coordinated		
				*x*	*y*	*z*
PCC / Precuneus	Both	824	–4.135	–3	–75	36
Striatum/Caudate /Insula	/	1149	5.2544	–24	15	0
Striatum	Left	/	5.2544	–24	15	0
Caudate	Right	/	3.6738	16	22	10
Insula	Left	/	4.2268	–33	19	–3
Insula	Right	/	3.4081	36	29	–1
Cerebellum	Left	716	4.7668	–12	–33	–39

**FIGURE 4:**
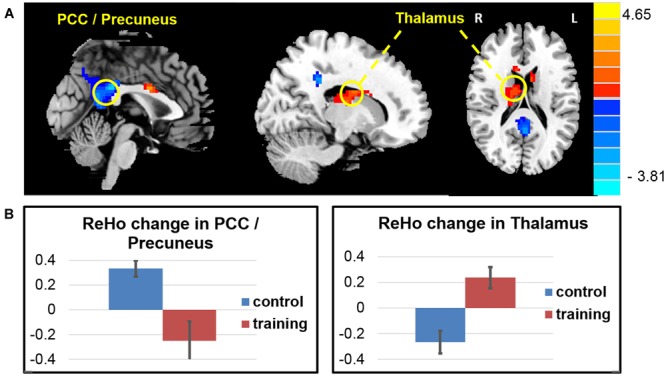
**ReHo changes after exercise training. (A)** The exercise training causes ReHo reduction in the PCC/precuneus areas and ReHo increase in a cluster covering the right thalamus and caudate, and the left middle frontal area. **(B)** Averaged ReHo *z*-score change of two groups in the PCC/precuneus areas and caudate.

Consistent with the findings in the ALFF, the whole brain voxel-wise ReHo analysis also revealed an decrease in the PCC/precuneus area and increase ReHo in the right thalamus and caudate, and the left middle frontal area (**Figure 4** and **Table 5**) in the exercise training group compared with the control group (two-sample *t*-test on the difference of two visits, *p* < 0.05, alphasim correction).

**Table 5:** Regions changed ReHo after 6 weeks (visit 2 – visit 1) in the exercise group compared with the control group.

Region	Hemisphere	Cluster size	Peak intensity	Peak MNI coordinated		
				*x*	*y*	*z*
PCC / Precuneus	Both	434	–3.8160	–12	–69	36
Caudate / Thalamus	/	173	3.7863	3	0	27
Caudate	Right	/	3.1527	18	–6	24
Thalamus	Right	/	3.1375	15	–9	18
Middle Frontal area	Left	299	4.6513	–21	27	33

**FIGURE 5:**
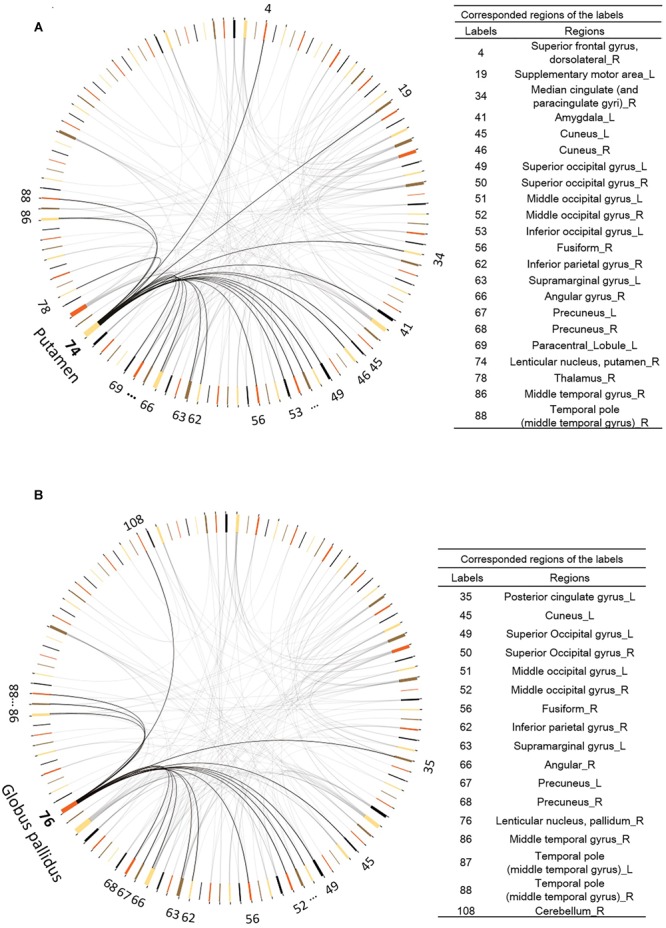
**Functional connectivity changes after exercise training. (A)** The right Putamen (AAL area 74) shows increased functional connectivity to other 21 regions. **(B)** The right Pallidum (AAL area 74) manifests increased functional connectivity to other 16 regions. Digit numbers represent the AAL labels.

Among the 116 ROIs from the AAL template, the right striatum (including the putamen (AAL area 74) and the globus pallidus (AAL area 76)) were identified because their changes in FC (visit 2 – visit 1) with other brain regions were significantly different between the two groups, and the number of significantly changed connectivity paths was ≥15, corrected for multiple comparisons at *p* < 0.05. As shown in **Figure 5A**, the right putamen increased connectivity with the many regions in the brain including the superior frontal gyrus, median cingulate, thalamus, amygdala, temporal cortex, occipital cortex, and parietal cortex (including the default mode regions such as the PCC/precuneus and inferior parietal regions). Similarly, the right globus pallidus (**Figure 5B**) increased connectivity with the temporal and occipital regions, as well as the PCC/precuneus post vs. pre exercise.

The graph theory analysis did not find any statistically significant difference in the global efficiency of any nodes (visit 2 – visit 1) between the exercise training group and the control group.

Because of the small sample size, we used the simple *t*-tests on change scores to increase power. We also used a mixed-effect model to validate the result. As shown in Supplementary Figures 1–3 in Supplementary Materials, our main results were confirmed by the analysis with the mixed-effect model.

### Correlations between the Neuroimaging Measures and Cognitive Function

**FIGURE 6:**
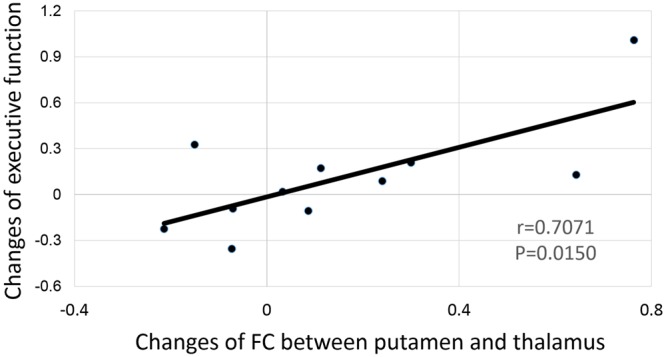
**Correlation between the functional connectivity change of the right putamen and the thalamus to the change of executive function**.

After corrected for multiple comparisons, we did not find any significant correlation between the changes in any neuroimaging measurements with changes in any measures on cognitive function. However, the changes of FC between the right putamen and right thalamus showed marginally significant correlation with the change of the executive function (*r* = 0.7071, *p* = 0.0150, **Figure 6**).

## Discussion

After this short 6-week period of physical exercise, our participants showed significant improvement in their executive and memory function. Across different measures examined in our study, the commonly found brain regions that showed significant changes after the 6-week exercise were the right striatum (including both the putamen and the globus pallidus) and the PCC/precuneus area. We noted a decrease in the striatum volume over the 6 weeks among the control non-exercise group. In comparison, the exercise training group showed no volume reduction in the right striatum, but increased functional connectivity between the right striatum and broad regions of the brain including the superior frontal gyrus, mid cingulate, amygdala, temporal, parietal (particularly the PCC/precuneus area), and the occipital cortices. This suggests an important role for exercise in preventing brain volume reduction, a phenomenon of brain aging. Importantly, the FC change between the right striatum and the right thalamus was marginally correlated with executive function. We integrated different measures to examine the training-induced neuroplasticity, and found that FC might be more sensitive among resting state functional activity/connectivity measures in evaluation of neuroplasticity related to cognitive improvement.

As a node of the cortico-striato-pallido-nigro-thalamo-cortical loop (DeLong et al., 1983), the striatum is well known for its role in motor planning, modulation of movement pathways (Alexander et al., 1986; Rolls, 1994), and a variety of cognitive processes including motivation and anticipation, procedural learning (Miyachi et al., 1997), as well as working memory (Voytek and Knight, 2010). As a supportive neural mechanism of the involvement of the striatum in exercise, increased metabolic capacity in the striatum has been found after 6-month exercise in rats (McCloskey et al., 2001). Increased brain-derived neurotropic factor (BDNF) levels in the striatum has also been reported after 6-week exercise in chronically stressed rats (Marais et al., 2009). FC analysis on the striatum by Di Martino et al. (2008) has also revealed that broad FC of the striatum with the rest of the neural systems is related to cognition, which is also consistent with our results. Therefore, it is not surprising that we found exercise could prevent volume loss and increase FC of the striatum to many other brain areas including the visual, temporal and default mode systems after exercise training, which could be related to the fact that all participants became skillful in the exercise programs they had practiced (based on the daily records of game performance level from the Wii console).

The other consistent change after exercise training occurred in the posterior cingulate cortex and precuneus area (PCC/precuneus). Although increased GM of the PCC/precuneus and increased FC between the PCC/precuneus and the striatum post exercise seems conflicting with the finding of reduced ALFF and ReHo in the PCC/precuneus, these results may not be hard to reconcile. Previous work has shown that the PCC is activated during mind wondering (Mason et al., 2007) and deactivated during meditation in mediators (Brewer et al., 2011). A recent real-time neurofeedback study has also shown that “undistracted awareness” such as “concentration” and “effortless doing” meditation has led to deactivation in the PCC (Garrison et al., 2013). It is possible that physical exercise can increase “effortless doing” ability with synchronized activity between the PCC and striatum and with low neural oscillation strength during resting state.

Although the current study focused on common findings among different imaging measures, we acknowledge that modality-specific results are also important because different measures reflect different neural mechanisms. For example, volumetric increase might be related to both neurogenesis and vasculogenesis/angiogenesis, whereas increased ALFF and ReHo during resting state should be related to increased amplitude or regional synchronization of automatic neural oscillations. We found significantly increased GM density in bilateral DLPFC, however, there was no significant changes in any of the functional measures in this region. It is possible that there was vasculogenesis/angiogenesis in the DLPFC post exercise without significantly enhancing neural function, or we can only find increased DLPFC activity when performing cognitive tasks but not during resting state. On the other hand, there was increased ALFF in the right entorhinal cortex without any volumetric changes. The entorhinal cortex is close to the hippocampus and critically involved in memory encoding (Eichenbaum and Lipton, 2008). However, the increased entorhinal ALFF did not correlate with cognitive performance, which could be related to the fact that our exercise training program involved in multiple tasks rather than memory specific. Future studies with exercise programs comparing how multiple task versus single learning task stimulate the hippocampal activation are necessary to further confirm the critical role of the hippocampus in physical exercise.

The significant increase in memory and executive function with 6-week exercise indicates that our mixed-domain exercise program is powerful and effective. Future studies in comparing of multi-domain versus single-domain exercise directly are necessary. It would also be interesting to further investigate whether the plasticity change of the striatum can be universally related to executive function in any exercise program, or if it is only specific to certain types of exercise.

As discussed above, small sample size, no control on different types of physical exercises, and lack of different training duration as comparisons are major caveats of the current study. While these caveats limited our major conclusions, we do believe this pilot study has several strengths. To our knowledge, this is the first study that compared different neural imaging measurements to study the neural plasticity and demonstrated that common regions exist in showing changes across different neuroimaging measures. Therefore, examining changes in multiple neuroimaging measures is recommended in longitudinal studies with a small sample size. In addition, this is one of the few that has found a robust improvement in both behavioral and neural function in a short training period.

## Conclusion

Our study demonstrated significant improvement in brain function as well as cognitive performance after 6 weeks of physical exercise training. Converging results indicate that our multi-domain exercise program could improve executive function through increasing function connectivity between the striatum and the thalamus. FC might be more sensitive among resting state functional activity/connectivity measures in evaluation of neuroplasticity related to cognitive improvement. In addition, our study suggests the effectiveness of multi-domain exercise training in improving cognitive function, and that identifying commonly changed regions across different imaging modalities could be an effective way to investigate neural plasticity.

## Author Contributions

LJ contributed in data analysis, interpretation of results, drafting the manuscript, approval for the final version for publication, and agreement to be accountable for the accuracy of all aspects of the work. HZ and Y-FZ contributed in data analysis, helped in interpretation of results and revising the manuscript, and approval for the final version for publication, and agreement to be accountable for the accuracy of all aspects of the work. GP and DS provided advice in study design and data collection, helped in interpretation of results and revising the manuscript, approval for the final version for publication, and agreement to be accountable for the accuracy of all aspects of the work. HG provided approval for the final version for publication. LW contributed in study design, data collection, helped in data analysis, interpretation of results, and revising the manuscript, approval for the final version for publication, and agreement to be accountable for the accuracy of all aspects of the work.

## Conflict of Interest Statement

The authors declare that the research was conducted in the absence of any commercial or financial relationships that could be construed as a potential conflict of interest.

## Footnotes

1 http://www.fil.ion.ucl.ac.uk/spm

2 http://dbm.neuro.uni-jena.de/vbm8

3 http://dbm.neuro.uni-jena.de/software/tom/

4 http://rfmri.org/DPARSF

5 http://www.fil.ion.ucl.ac.uk/spm/software/spm8/

6 http://www.nitrc.org/projects/gretna/
