# Supplementary material for: Multiple Neuroimaging Measures for Examining Exercise-induced Neuroplasticity in Older Adults: A Quasi-experimental Study
Source: Front Aging Neurosci. 2017 Apr 20;9:102. doi: 10.3389/fnagi.2017.00102 (PMC5397485; doi:10.3389/fnagi.2017.00102)
Supplement: Supplementary file 1 [file Presentation_1.PDF]

Supplementary Material

Multiple Neuroimaging Measures for Examining Exercise-Induced Neuroplasticity in Older Adults: a quasi-experimental study

Lanxin Ji[1], Han Zhang[2, 3], Guy G. Potter[4], Yu-Feng Zang[2, 3], David C. Steffens[5], Hua Guo[1], Lihong Wang[5,4,1*]

*** Correspondence:** Lihong Wang, MD, Ph D Email: lwang@uchc.edu

We've conducted repeated-ANOVA with SPM second level analysis (http://www.fil.ion.ucl.ac.uk/spm/doc/) on our neuroimaging data and found that the main results are similar to the t-test on changes. sFig.1 – sFig.3 are overlays of the ANOVA interaction effect results (blue) and the t-test results (red). Areas that showed significant changes in both of the tests are in purple.

1 Supplementary Figures

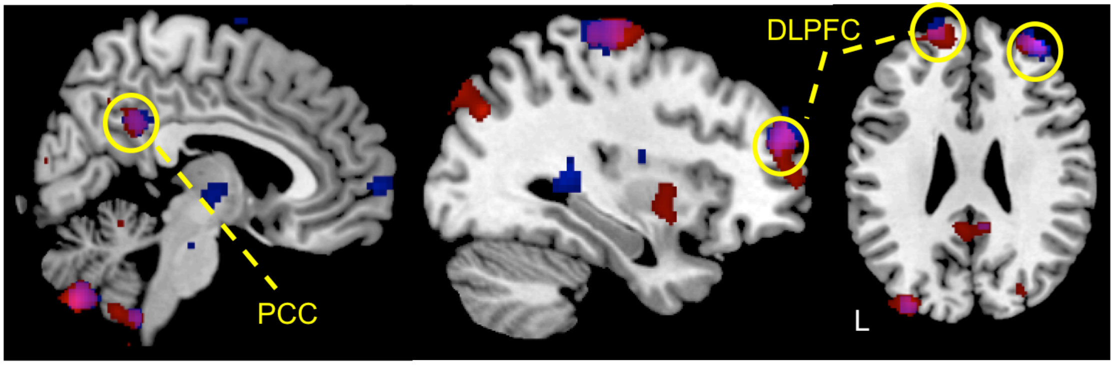

sFig.1 Grey matter volume changes. Both of the tests showed significant increase of grey matter volume in bilateral DLPFC, PCC, motor area, and cerebellum. There was no significant interaction effect in the striatum in the ANOVA analysis because only the control

group had gray matter reduction, but no change was found in the exercise group, which did not fit well with ANOVA interaction model. However, we believe that, as stated in the manuscript, prevention of grey matter reduction by exercise is important as well.

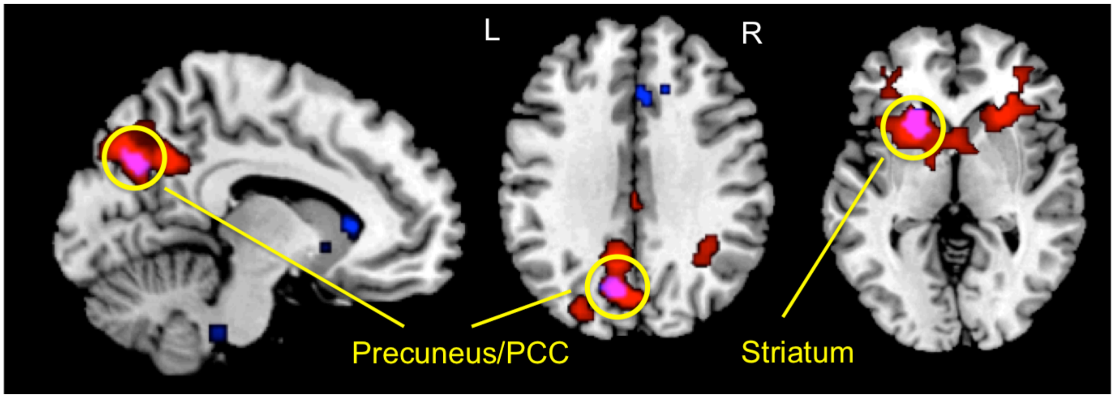

sFig.2 ALFF changes. As shown in the following figure, both tests showed that physical exercise has increased ALFF in left striatum and decreased ALFF in the PCC area.

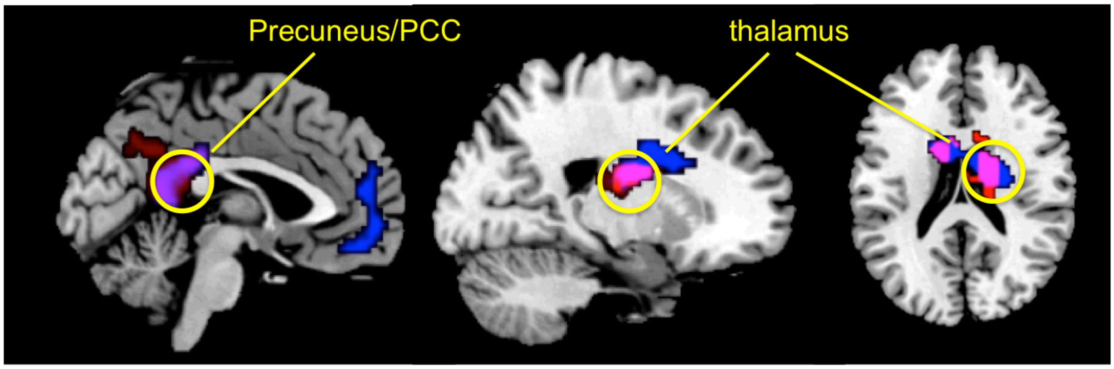

sFig.3 ReHo changes. Both of the tests showed exercise program has increased Reho in the thalamus and decreased Reho in the PCC area.
